# Effects of Sub-Lethal High Pressure Homogenization Treatment on the Adhesion Mechanisms and Stress Response Genes in *Lactobacillus acidophilus* 08

**DOI:** 10.3389/fmicb.2021.651711

**Published:** 2021-05-28

**Authors:** Giacomo Braschi, Margherita D’Alessandro, Davide Gottardi, Lorenzo Siroli, Francesca Patrignani, Rosalba Lanciotti

**Affiliations:** ^1^Department of Agricultural and Food Sciences, University of Bologna, Cesena, Italy; ^2^Interdepartmental Center for Industrial Agri-Food Research, University of Bologna, Cesena, Italy

**Keywords:** cell surface hydrophobicity, adhesion, *Lactobacillus acidophilus* 08, high pressure homogenization, moonlight protein, stress response, chaperonin, probiotics

## Abstract

Cell surface hydrophobicity (CSH) and adhesion are very important phenotypical traits for probiotics that confer them a competitive advantage for the resilience in the human gastrointestinal tract. This study was aimed to understand the effects over time of a 50 MPa hyperbaric treatment on the surface properties of *Lactobacillus acidophilus* 08 including CSH, autoaggregation, and *in vitro* adhesion (mucin layer and Caco-2 cells). Moreover, a link between the hurdle applied and the expression of genes involved in the general stress response (*groEL* and *clpP*) and adhesion processes (*efTu* and *slpA*) was evaluated. High pressure homogenization (HPH) at 50 MPa significantly increased the CSH percentage (H%), autoaggregation and *in vitro* adhesion on mucin of *L. acidophilus* 08 cells compared with the untreated cells. Moreover, the hyperbaric hurdle induced an upregulation of the stress response genes *groEL* and *ef-TU* together with a down regulation of the *clpP* and *S-layer slpA* genes. Looking at the protein profile, HPH-treatment showed an increase in the number or intensity of protein bands at high and low molecular weights.

## Introduction

Probiotics have become increasingly popular during the last decades as a result of the expanding scientific evidence that supports their beneficial effects on human health (FAO/WHO, 2001; Morelli and Capurso, 2012; Shewale et al., 2014). Most of the probiotic formulations contain lactobacilli, food-grade microorganisms widely applied in the food industry for their positive technological and health-promoting properties (Grosu-Tudor et al., 2016). Among them, *Lactobacillus acidophilus* is largely applied in the dairy sector and it is widely studied for its physiological, biochemical, genetic, and fermentative properties (Sanders and Klaenhammer, 2001; Weiss and Jespersen, 2010). Within this species, the probiotic *L. acidophilus* 08 (Burns et al., 2008), a strain already used as an adjunct for fresh cheeses and fermented milk preparations, can withstand very well cheesemaking and refrigerated storage conditions (Burns et al., 2008; Tabanelli et al., 2013). These technological characteristics, together with the functional ones, are fundamental when probiotics are meant to be incorporated in food. In fact, these beneficial microorganisms must be ingested at levels between 10^8^ and 10^10^ CFU/day to induce positive effects on the consumers. For this reason, several studies focused on identifying technological strategies which aim to improve probiotics viability during food processing and subsequent storage (Patrignani and Lanciotti, 2016). Among these strategies, the non-thermal technology based on high pressure homogenization (HPH) has already proved to enhance the survival of probiotic strains or to improve their overall functionality when treated in milk (Burns et al., 2008, 2015; Patrignani et al., 2009). Moreover, several studies showed that cell hydrophobicity, auto-aggregation, and *in vitro* resistance to simulated upper gastrointestinal tract were modulated in *L. acidophilus* 08 exposed to sub-lethal HPH treatments (50 MPa) (Tabanelli et al., 2013, 2014). In order to explain these changes, Tabanelli et al. (2015) proved that HPH is able to affect the outermost cellular stucture and induce a specific proteomic profile of the two probiotic strains *Lacticaseibacillus paracasei* A13 and *L. acidophilus* DRU, modulating in turns some of their technological and functional properties, including their ability to adhere and colonize the intestinal epithelium. For example, the higher interaction/adhesion observed of the *L. paracasei* A13 strain and intestinal epithelial cells upon hyperbaric treatment could be associated to cellular structure modifications (Tabanelli et al., 2012) involving the peptidoglycan structure, the different zwitterion character of the lipoteichoic acid (LTA), and surface proteins. Even the cell membrane, that contributes to separate microorganisms from the external environment, is considered one of the most susceptible targets of pressure and can respond in different ways to minimize sub-lethal stress (Serrazanetti et al., 2015). Changes in membrane composition are reflected in the modification of physical cell surface properties. According to the literature data, the presence of secondary cell wall polymers and adhesive proteins on the bacterial surface [i.e., mucin-binding proteins, fibronectin-binding proteins, surface layer proteins (Slps), surface layer associated proteins (SLAPs), and adhesion exoproteins] mediate the adhesion and immunomodulatory activities of *L. acidophilus* and other lactic acid bacteria (Palomino et al., 2016; Selle et al., 2017; Wang et al., 2018). For instance, the large amphiphilic interfacial polymer LTA plays pleiotropic roles in Gram-positive physiology and is a major immunomodulatory cell surface component. Even the S-layers proteins can act as a barrier against environmental hazards, control the transfer of nutrients and metabolites, maintain the cell shape, and promote surface recognition (Buck et al., 2005; Grosu-Tudor et al., 2016). However, SlpA, the major component of the S-layer in *L. acidophilus*, contributes to epithelial cell-bacteria interaction at the gut level and to the immune response modulation.

Although literature data are consistent in reporting the positive effects of sub-lethal HPH treatments on the probiotic features (i.e., hydrophobicity and autoaggregation) in a strain dependent way, little is known about the microbial gene expression upon this stress. Recently, Siroli et al. (2020) showed that *L. paracasei* A13 activated a series of reactions which aimed to control and stabilize membrane fluidity in response to HPH treatments inducing an upregulation of genes involved in fatty acids biosynthesis as an immediate response mechanism adopted by *L. paracasei* A13 to HPH. However, information is scarce following the application of HPH sublethal treatment on several stress response proteins, such as the chaperonins GroEL and DnaK, proteases ClpP, GroEL, and DnaK, which play a key role in several stress conditions (Frees et al., 2007; Weiss and Jespersen, 2010) and the genes involved in microbial cell adhesion in *L. acidophilus*.

Thus, in this framework, the present study investigated the effects of a sub-lethal HPH treatment (50 MPa) on the different phenotypical traits involved in the adhesion phenomena processes like cell surface hydrophobicity (CSH), autoaggregation, and *in vitro* adhesion (both in mucins and Caco-2 cells) of *L. acidophilus* 08, a probiotic strain commonly used in commercial functional dairy products. Structural modifications induced by the sub-lethal HPH treatments were observed by transmission electronic microscope. Moreover, the up or down regulation of genes involved in the general stress responses (*groEL* and *clpP*) and adhesion processes (*efTu* and *slpA*) were assessed, as well as the overall protein profile of the treated and untreated *L. acidophilus* 08 cells.

## Materials and Methods

### Bacterial Strain

*Lactobacillus acidophilus* 08, a commercial probiotic strain isolated from dairy Argentinean dairy products, was stored at −80°C. Before the experiments, *L. acidophilus* 08 was cultured three times without stirring, for 24 h in de Man, Rogosa, and Sharpe (MRS) broth (Thermo Fisher Scientific, Milano, Italy) at 37°C.

### High-Pressure Homogenization Treatments

*Lactobacillus acidophilus* 08 cells after 18 h at 37°C of growth (early stationary phase cells) in MRS broth were subjected to HPH at 50 MPa using a PANDA plus 2000 high-pressure homogenizer equipped with heat exchanger (Niro Soavi, Parma, Italy). Sample inlet temperature was 25°C, while after treatment the sample temperature was approximately 30°C. After the hyperbaric treatment, samples were taken for the gene expression and CSH assays as described below.

### Phenotypical Trials

#### Cell Surface Hydrophobicity Kinetics

The CSH of *L. acidophilus* 08 cell was evaluated as hydrophobicity percentage index (H%) according to Vinderola and Reinheimer (2003), with some modification as suggested by Tabanelli et al. (2013).

The ability of the strain considered to adhere to n-hexadecane and its evolution during 2 h was tested in relation to the HPH treatment applied (50 MPa). The H% index was calculated with the formula:

$$H\%=\left(\frac{\mathrm{A0}-\mathrm{At}}{\mathrm{A0}}\right)*100$$

where At represents the absorbance at 560 nm after 1 h of incubation at 37°C, while A0 represents the initial absorbance (Vinderola and Reinheimer, 2003; Tabanelli et al., 2013). After the HPH treatment at 50 MPa, *L. acidophilus* 08 cell suspension was centrifuged at 8,000 rpm for 10 min at 4°C, washed two times with NaCl 0.9% isotonic solution, and then, the cell optical density (OD) at 560 nm was adjusted with the same solution to 1.

#### Autoaggregation

Autoaggregation assay was performed as described by Valeriano et al. (2014). After HPH treatment, the treated and untreated cells were harvested by centrifugation at 3,800 rpm for 10 min at 10°C, washed twice, and resuspended in sterile PBS to obtain approximately 10^8–9^ CFU/mL viable counts. Cell suspensions (40 mL) were mixed by vortexing for 10 s and, autoaggregation was determined after three and 6 h of incubation at room temperature. To measure the autoaggregation, 100 μL of the upper suspension were transferred to 900 μL of PBS and the absorbance (A) was measured at 600 nm. The autoaggregation percentage was expressed as:

$$\%\mathrm{Autoaggregation}=1-\left(\frac{{\text{A}}_{\text{t}}}{{\text{A}}_{0}}\right)*100$$

where At represents the absorbance after 3 and 6 h of incubation, while A0 is the absorbance at t0.

#### Adhesion Assay to Mucins

The assessment of microbial adhesion to mucins was performed following the protocol of Valeriano et al. (2014) with some modifications. 96-well polystyrene microtiter plates (Sigma, Bornem) were used to obtain a high-throughput adhesion assay. Mucous gel that covers the intestinal epithelium was simulated by mixing porcine mucin type II (Sigma) (5%, w/v) and agar (0.8%) resuspended in phosphate-buffered saline (PBS, pH = 7.0) according to Van den Abbeele et al. (2009). Mucin agar was sterilized by autoclaving at 121°C for 15 min and, then, 100 μl were added in each well. Approximately 100 μl of bacterial suspension (∼10^8–9^ CFU/ml) were washed, suspended in PBS buffer (pH 7.0), and added to the wells. Plates were incubated at 37°C for 1 h. After incubation, wells were washed five times with 200-μl sterile citrate buffer (pH 5.9) to remove unbound bacteria. Another 200 μl of 0.5% (v/v) Triton X-100 was then added to isolate the attached bacteria. The viable cell count, expressed as CFU/ml, was determined in all cases by plating on the MRS media. Each assay was performed in quadruplicate. Percentage adhesion was calculated as follows (Collado et al., 2008):

$$\%\mathrm{Relative}\mathrm{Adhesion}=\left(\frac{\text{CFU}/\mathrm{mL}\,\mathrm{after}\,\mathrm{adhesion}}{\text{CFU}/\mathrm{mL}\,\mathrm{before}\,\mathrm{adhesion}}\right)*100$$

The assay was performed with HPH treated cells incubated for 60, 90, and 120 min and 24 h before the adhesion test, and untreated *L. acidophilus* 08 and *Lactobacillus rhamnosus* GG as controls.

#### Adhesion Assay to Caco-2 Cells

In order to describe the ability of *L. acidophilus* 08, treated at 50 MPa, to adhere to the intestinal epithelium, Caco-2 cell line was used. This cell line is derived from human colorectal adenocarcinoma and presents the ability to differentiate into cells with many properties typical of enterocytes (Lea, 2015). Caco-2 cells were routinely grown in DMEM high glucose medium (Sigma, Milan, Italy) with the addition of 2 mM L-glutamine (Sigma, Milan, Italy) and 20% v/v Bovine Fetal Serum (Sigma, Milan, Italy) in flasks for cell cultures (Corning, NY, United States), at 37°C with 5% CO_2_. To obtain the differentiated Caco-2 cultures, the cells were inoculated at a density of 10^5^ cells/cm^2^ and kept in culture for 21 days, changing the culture medium every 3–4 days. For adhesion tests, Caco-2 cells were inoculated on sterile glass coverslips in six-wells plates and grown until differentiated. Cells were then incubated with exponentially growing lactobacilli cells by applying a 1:400 ratio, at 37°C with 5% CO_2_ for 1 h, and washed twice with PBS to remove the non-adherent lactobacilli. Samples were then fixed with methanol for 10 min and stained with Giemsa 10% (Sigma, Milan, Italy) for eight min. Afterward, samples were washed three times with PBS, and then air dried and observed by an optical microscope (1000× magnification). Also, the untreated cells of *L. acidophilus* 08 were tested. Adhesion to Caco-2 cells was evaluated by counting the number of adherent *Lactobacillus* cells to Caco-2 cells, considering at least 200 Caco-2 cells.

#### Transmission Electronic Microscope

Transmission electron microscopy (TEM) was used to investigate the morphological changes caused by the HPH treatment. Ten milliliters of the control samples and the HPH-treated samples were centrifuged (8,000 *g*, 10 min) and the pelleted cells were fixed by suspending them in 2.5% glutaraldehyde (in 0.1 M PBS buffer, pH 7). These samples were stored at 4°C for 2 h. After aldehyde fixation, the samples were prepared according to Bury et al. (2001). The post-fixed cells were washed using the same buffer, and then, they were dehydrated for 15 min using the following series of ethanol solutions: 50, 75, 90, and 100%. The dehydrated cells were infiltrated with increasing concentrations of Spurrresin (Agar Scientific, Stansted, Essex, United Kingdom) over 24 h. Polymerization of the resin was achieved by heating the samples in an oven at 65°C for 18 h. These sections (∼90 nm thick) were placed on carbon-coated Form var-covered 300-mesh copper grids for approximately 15 min, rinsed using 20 drops of distilled water, negatively stained using 6–7 drops of 2% aqueous uranyl acetate, and then, examined using a Philips CM10 transmission electron microscope.

### Reverse Transcription Quantitative PCR

*Lactobacillus acidophilus* 08 RNA was extracted using the MasterPure^TM^ Complete DNA and RNA Purification (Lucigen). The yield and the purity of each extraction was determined by measuring the absorbance at 260_nm_ and 280_nm_ using a BioDrop μLITE (BioDrop, Milan, Italy). For all the samples, the yields were about 700 ng/μL and only samples with a ratio 260_nm_/280_nm_ between 1.9 and 2.1 were used for the reverse transcription reaction.

The reverse transcription into cDNA was performed using the Reverse Transcription System Kit (Promega, Wisconsin, WI, United States) following the manufacturer’s instruction. Before the real time assays, samples were properly diluted in DNAse/RNAse free water (Promega, Wisconsin, WI, United States) to reach a final concentration of 5 ng/μL.

Reverse transcription quantitative PCR (RT-qPCRs) were performed using a Rotor gene 6000 thermal cycler (Corbett Life Science, Mortlake, NSW, Australia). The list of genes and their function are reported in Table 1. The best RT-qPCR reaction conditions for each primer stet were investigated by end point PCRs using *L. acidophilus* 08 genomic DNA as a template. Different final concentrations of MgCl_2_ (2.00, 3.00, 4.00 mM) and annealing temperatures (AT) were tested. Amplification quality was verified by gel electrophoresis using 1.5% agarose gels (data not showed).

**TABLE 1:** List of genes and primers and protein functions considered in this trial.

Gene	Primer 5′ > 3′	Protein function	Ta^1^ (°C)	References
*groEL*	F: GCTGTTAAGGCACCTGGTTTTG	Molecular chaperonins	60	Weiss and Jespersen, 2010
	R: AAGGGCTGCAATGTCTTCAAG			
*efTU*	F: GGTGCTATCTTAGTTGTTGC	Translational elongational factor TU	55	Ramiah et al., 2009
	R: CAACCAAGTCGATCAATTCT			
*slpA*	F: GCACAACGCATACTACTACG	S-layer protein A	55	Ramiah et al., 2009
	R: CTTGTCAACAGCCTTACCGT			
*clpP*	F: GCAATCGGTATGGCAGCAT	ATP-dependent Clp protease	60	Weiss and Jespersen, 2010
	R: ACGCTTACCCTTTGTACCACTTG			
*gadpH (RG)*	F: CTATCGTTTACTCAGTAAACCAAGA	Glyceraldehyde-3-phosphate dehydrogenase	55	Ramiah et al., 2009
	R: CGTGGATAGTAGTCATAGTACCAAC			
*23S rRNA (RG)*	F: TGTCAGGTGGGCAGTTTGAC		60	Weiss and Jespersen, 2010
	R: TTGAGCGCCTCCGTTACAC			

*^1^Annealing temperature (°C).*

The RT-qPCR reaction mixture (10 μL) included 5 ng of cDNA, 10 μL of SsoFast EvaGreen Supermix (Bio-Rad), 500 nM each primer, and 8 μL DNAse/RNAse free water (Promega, Wisconsin, WI, United States). Each reaction was performed in triplicate.

For each gene, a threshold line and a quantitative cycle (Cq) were determined using the Rotor-Gene series software (Qiagen Inc., Ontario, ON, Canada). For each primer pair, the amplification efficiency (E) was calculated as described by Pfaffl (2001) using the formula:

$$\text{E}=10^{\left(-1/\text{slope}\right)}$$

and genomic *L. acidophilus* 08 DNA standard curves built using five different DNA concentrations.

### Relative Gene Expression Analysis

The relative gene expressions indexes (RGE indexes) were determined according to the MIQE guidelines (Bustin et al., 2009) using the mathematical model proposed by Pfaffl (2001). Based on literature, the performance of two Reference Genes (RGs) were tested in the experimental condition: *23S RNA* and *gadpH*. The gene expression stability of the two candidates RGs were analyzed using the BestKeeper© tool program (Pfaffl et al., 2004), and on the basis of the Pearson correlation index, *23S RNA* was chosen.

### *L. acidophilus* 08 SDS-PAGE Protein Analysis

Total *L. acidophilus* 08 proteins were extracted from the HPH treated and untreated cells according to Palomino et al. (2016) with some modifications. From each sample, 20 mL of *L. acidophilus* 08 suspension was collected and washed with sterile PBS (pH 7.0) by refrigerated centrifugation (10°C) at 3,800 rpm for 10 min. Harvested cells were diluted again in PBS buffer until an OD 600_nm_ = 1.0 was reached. Concentrated cell suspension was diluted 1:1 in loading buffer [20% glycerol, 4% sodium dodecyl sulfate (SDS), 1% β-mercaptoethanol, Tris–HCl 0.15 M] and total proteins were extracted by heating at 100°C for 5 min. After, the incubation protein samples were analyzed by electrophoresis on 8–16% SDS-PAGE gels (Biorad, Milan Italy). Electrophoresis was performed at a constant voltage of 100 V for 30 min and then at 200 V until the run was completed.

### Statistical Analysis

Data were processed using the STATISTICA 8 software tool (Version 8.0; Statsoft., Tulsa, OK, United States).

The CSH, cell loads, and relative gene expression (RGE) detected in relation to the 50 MPa homogenization pressure applied and to the frame time considered (2 h) were considered significant (*p < 0.05*) on the basis of the ANOVA and TUKEY HSD *post hoc* test, while adhesion to mucus and autoaggregation were significant (*p* < 0.05) on the base of the *t*-test. For both tests, the untreated control cells were considered the control condition.

## Results

### Effects of HPH at 50 MPa on *L. acidophilus* 08 Cell Viability and Cell Surface Hydrophobicity

**FIGURE 1:**
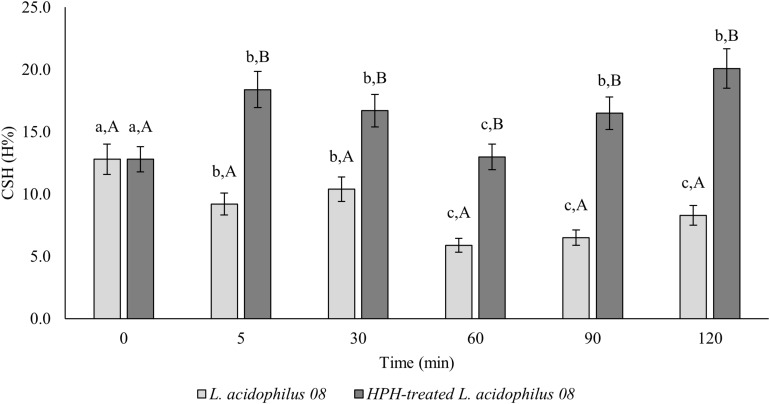
*Lactobacillus acidophilus* 08 cell surface hydrophobicity (CSH) (H%) of the untreated (*L. acidophilus* 08) and treated cells at 50 MPa (HPH-treated *L. acidophilus* 08). The results are the average of three independent replicates (*n* = 3). Error bars indicate the SD. Among the series, different letters indicate the samples that are significantly different (*p* < 0.05). Different lowercase letters indicate the significant differences for a considered sample during the frame time considered (0–120 min), while capital letters indicate the significant differences at the considered time differences between samples (untreated control and treated).

In Figure 1, the changes in CSH of *L. acidophilus* 08 (H%) after the homogenization treatment at 50 MPa are reported. The H% of treated *L. acidophilus* 08 increased up to 18.4% after 5 min of incubation, then it decreased in the following 60 min, and it increased again during the remaining period considered (120 min). On the contrary, the H% of untreated cell decreased from 10 to 5% over time (Figure 1). The increased hydrophobicity observed in the treated samples was not associated with a reduction of *L. acidophilus* 08 viability. In fact, as shown in Table 2, no significant loss of cell viability (*p* < 0.05) (Log CFU/mL) was observed upon HPH treatment within the following 120 min of incubation.

**TABLE 2:** Viability of the untreated (control) and treated (HPH at 50 MPa) cells of *L. acidophilus* 08 over time (0–120 min).

	*Log CFU/mL*	
Time (min)	Untreated control	50 MPa
0	8.53^a,A^ ± 0.23	8.85^a,A^ ± 0.18
5	8.69^a,A^ ± 0.47	8.29^a,A^ ± 0.26
30	8.44^a,A^ ± 0.38	8.41^a,A^ ± 0.42
60	7.74^a,A^ ± 0.31	7.99^a,A^ ± 0.24
90	8.25^a,A^ ± 0.38	8.37^a,A^ ± 0.45
120	8.21^a,A^ ± 0.31	8.13^a,A^ ± 0.30

*Different lowercase letters indicate significant differences among control and treated samples. For each considered sample, different capital letters indicate significant differences along the time.*

### Effects of HPH at 50 MPa on *in vitro* Adhesion of *L. acidophilus* 08 in Mucin and Caco-2 Cells

**FIGURE 2:**
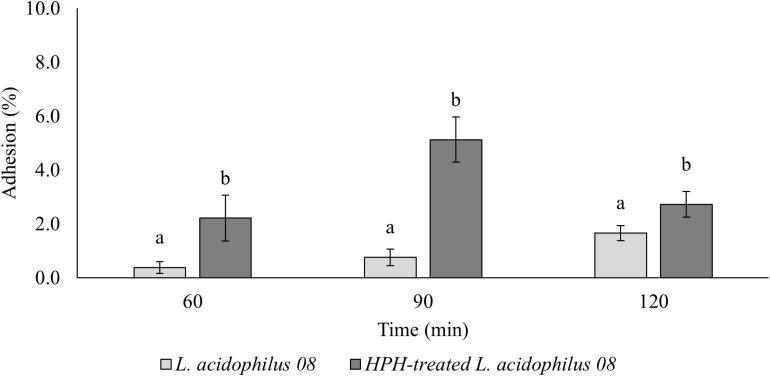
*Lactobacillus acidophilus* 08 percentage adhesion values of the untreated (control) and treated (HPH at 50 MPa) cells over time (60–120 min). Relative adhesion was calculated on the base of CFU/mL before and after the adhesion assay. The results are the average of four independent replicates (*n* = 4). Error bars indicate the SD. Different lowercase letters indicate the significant differences (*t*-test; *p* < 0.05) for a considered sample during the frame time considered (120 min).

The HPH at 50 MPa increased the mucin adhesion properties of *L. acidophilus* 08 (Figure 2). In fact, while the control had a relative adhesion ranging between 0.38 and 1.66%, in HPH-treated cells, however, this value increased up to 2.22 and 5.13% after 60 and 90 min, respectively, and then, it reduced to 2.73% at 120 min (Figure 2). The same trial was performed assessing the adhesion of the treated and untreated bacteria on Caco-2 cells. In this case, only the time point that showed the best mucin adhesion (90 min) was tested. However, no significant differences were observed (9.49 ± 4.88 and 7.42 ± 2.48 adherent *L. acidophilus* 08/Caco-2 cells for untreated and HPH-treated samples, respectively).

### Effects of HPH at 50 MPa on Autoaggregation of *L. acidophilus* 08

**FIGURE 3:**
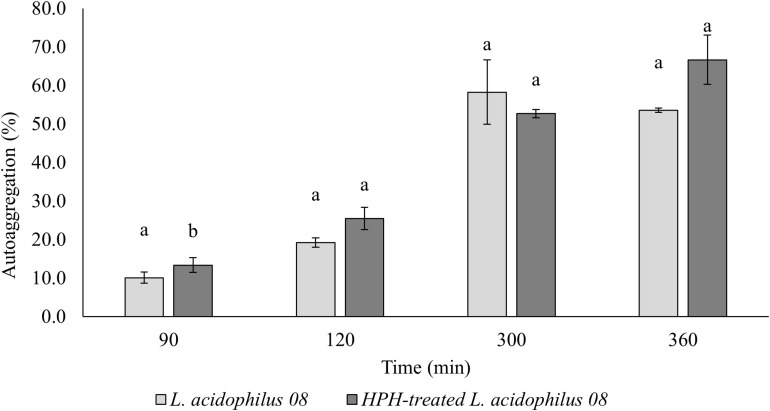
*Lactobacillus acidophilus* 08 percentage autoaggregation values of the untreated (control) and treated (HPH-treated *L. acidophilus* 08) cells over time (90–360 min). Autoaggregation was calculated on the base of optical density (OD) at λ = 600_nm_ before and after incubation from HPH at 50 MPa treatments. The results are the average of three independent replicates (*n* = 3). Error bars indicate the SD. Different lowercase letters indicate significant differences (*t*-test; *p* < 0.05) for a considered sample during the frame time considered.

In Figure 3, the autoaggregation kinetic over time (90–360 min) of the treated and untreated *L. acidophilus* 08 is reported. Significant differences (*p* < 0.05) between the two samples were obtained after 90 and 120 min, with a higher autoaggregation in the HPH-treated cells that reached values of 25.45% after 120 min. On the contrary, no differences were observed after 300 and 360 min (Figure 3).

### Effect of HPH at 50 MPa on the Outermost Cellular Structure of *L. acidophilus* 08

**FIGURE 4:**
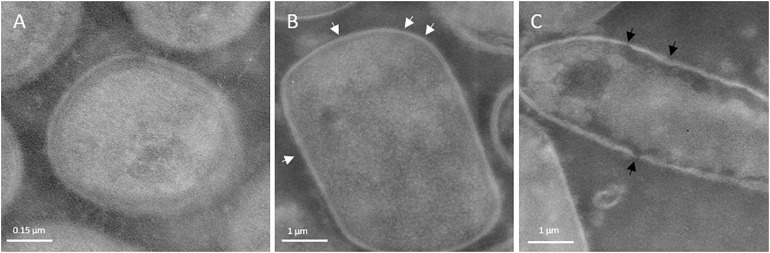
Transmission electron micrographs of *Lactobacillus acidophilus* 08: untreated control **(A)**; 50 MPa HPH treated cells **(B,C)**. White arrows indicate discontinuous and fragmented surface; black arrows indicate cell wall sublethal damages. Magnification:52,000×.

Figures 4A–C show the TEM images of the treated and untreated cells of *L. acidophilus* 08, a species commonly characterized by the presence of an S-layer in the cell envelope. Immediately after the treatment, HPH impacted on the outermost cellular structures. In fact, the continuous and tinny S-layer of untreated *L. acidophilus* 08 (Figure 4A) was modified generating a discontinuous and fragmented surface in the HPH-treated cells (Figure 4B). Moreover, some sublethal damages were observed in the cell wall due to the mechanical stress imposed (Figure 4C).

### Effects of HPH at 50 MPa on the Adhesion and Stress Response Genes of *L. acidophilus* 08

**FIGURE 5:**
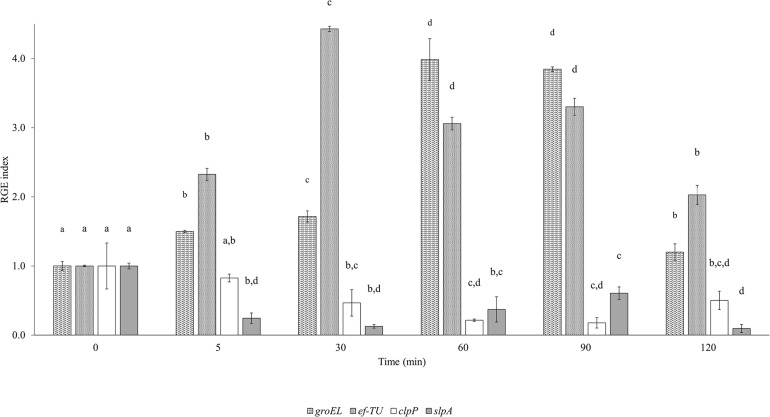
Evolution of the Relative Gene Expression (RGE) index of Lactobacillus acidophilus 08 groEL (60 kDa chaperonin), ef-TU (elongation factor-TU), clpP (ATP-dependent Clp protease proteolytic subunit), and slpA (S-layer porotein A) genes after high pressure homogenization at 50 MPa using 23S RNA as the reference gene. Results are the mean of three independent replicates (*n* = 3) collected in a time frame of 0–120 min. Error bars indicate the SD. Different letters are referred to the significant differences (*p* < 0.05) of the RGE of each gene during the frame time considered.

**FIGURE 6:**
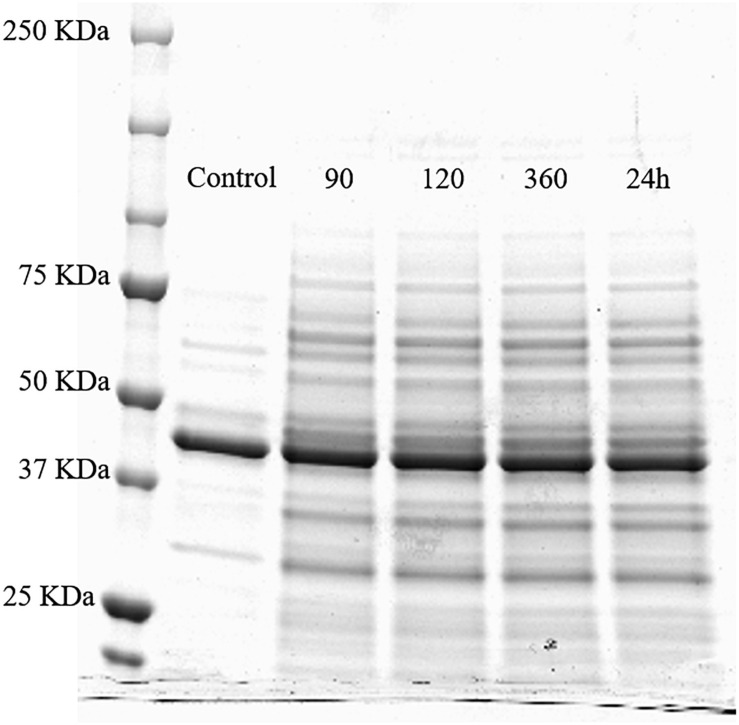
SDS-PAGE profile of whole cell proteins (500 μL OD_600 nm_ = 10) of the untreated *Lactobacillus acidophilus* 08 (control) and HPH treated cells after 90, 120, and 360 min, and 24 h upon the treatment.

Figure 5 illustrates the expression of adhesion (*efTU* and *slpA*) and acid stress response (*groEL* and *clpP*) related genes in the 120 min that followed the hyperbaric treatment (Figure 5). *groEL* and *efTU* were upregulated, while *clpP* and *slpA* were downregulated (Figure 6). In particular, *groEL* and *ef-TU* showed a similar expression trend within the first 5 min, with a 1.5 and 2-fold increase (*p* < 0.05), respectively. Then, the gene induction wave reached a maximum of fourfold increase between 30 (*ef-TU*) and 90 (*groEL*) min and eventually decreased to values that are close to the initial ones at 120 min (*p* < 0.05) (Figure 2). On the contrary, homogenization at 50 MPa significantly (*p* < 0.05) reduced the expression of *clpP* and *slpA* in the 2 h following the treatment. A half-fold reduction in *clpP* expression was reached after 30 min from the treatment and the down regulation persisted for 2 h (Figure 5). A similar behavior was observed for *slpA* (Figure 5).

### Effects of HPH at 50 MPa on Whole Cell Proteins of *L. acidophilus* 08

The separation of the proteins extracted from *L. acidophilus* 08 after HPH at 50 MPa and during the next 24 h was carried out by the SDS-PAGE (Figure 6). Proteins of the untreated *L. acidophilus* 08 were taken as the control. Protein bands were estimated by the comparison with the molecular mass standards (20–250 KDa). Compared with the control, hyperbaric treatment samples showed a higher number of proteins bands (and in some cases more intense), having low and high molecular weights, even after 90 min from the hurdle. This effect was kept in the subsequent 24 h (Figure 6).

## Discussion

In accordance with the literature, sub-lethal levels of HPH represent a useful tool to increase the probiotic features in a strain dependent manner. In the present research, the hyperbaric treatment performed at 50 MPa modulated the phenotypical cell surface traits of *L. acidophilus* 08 without effecting its viability. In fact, HPH increased the CSH and autoaggregation over time. Compared to the untreated bacteria, HPH treated cells increased their CSH up to 20% for the entire 120 min considered, while autoaggregation differences were observed only up to 90 min from the start of the treatment. Regarding the adhesion assays tested, no differences were observed in the Caco-2 cells. However, this result could have been affected by the single time point tested and the fact that Caco-2 are non-mucus producing cells (Gagnon et al., 2013). In fact, on the trial performed on a mucin layer, the treated bacteria showed an improved adhesion compared with the untreated ones (5.13 and 0.76 %, respectively, after 90 min from the hyperbaric hurdle). The collected data seem to prove that the response of *L. acidophilus* 08 to HPH is related to a modulation of its adhesion during a timeframe that goes from 60 to 120 min upon the treatment. These results can be considered a further advance compared with those described by Tabanelli et al. (2013) where hydrophobicity and autoaggregation were evaluated only immediately after HPH treatment. However, the differences in the two studies concerning the initial values of cell hydrophobicity and autoaggregation could be explained by the different media applied and cell growth stage. In fact, according to Klotz et al. (2017), the S-layer of *L. acidophilus* changes between the logarithmic and early stationary phases. Moreover, preculturing and inoculating methods could have a strong impact on cell behavior in relation to the experimental conditions applied (Kragh et al., 2018). TEM results were consistent with the findings of Tabanelli et al. (2015) regarding *L. acidophilus* DRU treated with HPH. In fact, the hurdle generated a discontinuous and fragmented cell surface. In the present study, sublethal damages of the cell wall due to the mechanical stress imposed were also observed. However, as already reported by Tabanelli et al. (2013), HPH did not decrease *L. acidophilus* 08 viability. The barotolerance of lactic acid bacteria such as *Lactiplantibacillus plantarum* subsp. *plantarum*, *L. paracasei* A13, and *L. acidophilus* Dru was already described by Lanciotti et al. (2007) and Tabanelli et al. (2013) using sub-lethal pressure. Even a higher pressure (130 MPa) showed a scarce reduction in the viability of *Lactobacillus helveticus* and *L. plantarum* subsp. *plantarum* strains (Vannini et al., 2004) confirming this barotolerance.

The comprehension of the genetic mechanisms regulating the CSH is very important when studying probiotics since they can be correlated with the microbial capacity to adhere and, to some extent, colonize the intestinal epithelium, offering a competitive advantage to the selected strain (Schillinger et al., 2005). The data regarding gene expression suggested that the hyperbaric treatment was able to specifically modulate *groEL* and *clpP*, which are both associated with the general stress response mechanisms. *groEL* codes for a cytoplasmic chaperonin protein (GroEL) and it is widely conserved among microbial species. Together with GroES and DnaK, GroEL is part of the repair complex for the folding of denatured polypeptides, and it is involved in many stress responses (Weiss and Jespersen, 2010; Hernández-Alcántara et al., 2018) such as the exposure to acids and bases, high saline concentrations, ethanol, oxygen, and heat (Hartl, 1996; Wickner et al., 1999; Saibil, 2008). In fact, during these conditions, chaperonin proteins are expressed and they revert the denaturation of unfolded polypeptides (Weiss and Jespersen, 2010; Mbye et al., 2020). When chaperonins fail to fold the damaged proteins, the molecular scavenger ATP-dependent Clp protease acts in degrading the damaged proteins. In our experiment, the HPH treatment produced a significant overexpression of *groEL* that was maintained for 90 min, after that, it started to decline going back to the starting level.

Compared to *groEL*, the protease gene *clpP* showed an opposite response. In fact, the HPH treatment reduced its expression during the following 120 min of incubation. Thus, this may indicate that the upregulation of *groEL* pass through an alternative induction pattern not associated with the general stress response. The data of the present research are partially in agreement with those found by Weiss and Jespersen (2010) on *L. acidophilus* NCFM during a gastrointestinal simulated digestion where the gastric conditions had a significant inducive effect on *groEL* and a moderate impact on *clpP*. This suggests that the HPH treatment may lead to a gene expression in *L. acidophilus* 08 like the one induced by acid stress.

Although GroEL chaperonin is predominantly intracellular (Gupta, 1995), some authors have identified the presence of this protein on the cell surface of different probiotics and pathogens (Kainulainen and Korhonen, 2014) where it becomes part of the secretome. This complex gives the strains an advantage for ecological niche colonization. Moreover, other studies have suggested that *groEL* has a moonlight behavior (Kainulainen and Korhonen, 2014; Amblee and Jeffery, 2015; Jeffery, 2019), that is, the capability to perform more than one biological function, including adhesion (Kainulainen and Korhonen, 2014). As adhesine like protein, groEL can promote cell adhesion to mucus and epithelial cells, stimulate cytokine production, and inhibit enteropathogen adhesion to the host cell surface (Bergonzelli et al., 2006; Ramiah et al., 2008; Sánchez et al., 2009; Spurbeck and Arvidson, 2012; Sun et al., 2016; Peng et al., 2018). As reported by literature, the elongation factor-Tu (EF-Tu) should also have a moonlight behavior. In fact, other than functioning as a G-protein that facilitates the correct transfer of aminoacyl-tRNA to ribosomes during protein synthesis (Gaucher et al., 2001), EF-Tu can have an adhesion-like function (Granato et al., 2004; Amblee and Jeffery, 2015; Jeffery, 2019). Like GroEL, EF-Tu enhanced the adhesion of *Lactobacillus johnsonii* NCC 533 (La1) to the intestinal mucosa (Granato et al., 2004; Bergonzelli et al., 2006). In our study, *ef-TU* was induced by the sublethal HPH treatment and the pattern reflected the one observed for *groEL*, with the maximal expression level reached after 60–90 min. Moreover, Ramiah et al. (2009) found that simulated gastro duodenal environment (MRS broth supplemented with mucine, bile salt, and pancreatin) induced 40-fold the expression of the *efTU* gene in *L. acidophilus* ATCC 4356. This may suggest that HPH treatment had a similar impact on *L. acidophilus* 08.

Although GroEL and EF-Tu are recognized for their moonlight effect, it cannot be inferred that their increased gene expression upon sublethal HPH treatment also determines a better adhesion. For sure, their increase is a response to stress conditions. In fact, during the passage through the homogenizing valve, millisecond increases of temperature and a rise in gas partition into cytoplasmic membranes generated a marked oxidative stress for the cells that can be counteracted more or less effectively, depending on the microbial species, strains, and protocols applied (Siroli et al., 2020). Although, this is particularly true when cells are subjected to 150 and 200 MPa, it becomes minimal when a reduced pressure is applied, as in our case (50 MPa).

To better clarify the surface modifications, *slpA* gene expression was investigated. SlpA protein represents one of the three major proteins of the outermost cell envelope S-layer in *L. acidophilus* (Johnson et al., 2013). S-layer is a crystalline matrix of non-covalent bounded proteins (Slp proteins) that affects the surface properties of several lactobacilli including dairy-fermenting and mucosal associated strains (i.e., *L. acidophilus, Lactobacillus crispatus*, and *Lactobacillus hevleticus*) (Hynönen and Palva, 2013). The HPH at 50 MPa induced a strong down regulation of the *slpA* gene immediately after the treatment and during the following 120 min considered. However, this reduction was not associated with a decrease in CSH, or other phenotypic traits such as autoaggregation or adherence to mucin. This could suggest that the mechanisms regulating the adhesion of *L. acidophilus* 08 could also be associated to a positive modification of the other outermost structure such as the cell wall and LTA. In fact, Fina Martin et al. (2019) demonstrated that LTA serves as an anchor for the S-layer protein and anchor the S-layer to the lactobacilli cell wall. Even Iucci et al. (2007) hypothesized that HPH treatment might induce an increased exposure of the hydrophobic regions of proteins, in relation to the level of treatment. This hydrophobicity seemed to be a key factor for the enhanced antimicrobial action of HPH treated enzymes such as lysozyme or lactoferrin. It has been reported that pressure can disturb the large supramolecular structure of proteins, allowing the single components to move freely, and become independent from the original structure. Interactions could be restored when the pressure instantaneously decreases, however, the original structure was not maintained because of the independent movements of the components (Iucci et al., 2007). According to Palomino et al. (2016), SlpA has a MW of 45.9 kDa and represents 90% of the S-layer proteins in *L. acidophilus* ATCC 4356. In our work, the untreated cells also possessed a very thick band in SDS-PAGE that could correspond to SlpA. This band did not change upon HPH treatment. The discrepancy between gene downregulation and presumptive protein abundance is not unknown. In fact, gene expression does not always reflect the protein amount because of the various levels of regulation inside the cells (Liu et al., 2016). Moreover, in our case, the decrease observed was around 1 RGE, a value maybe not enough to be observed from a protein content point of view. Another possible hypothesis is that an S-layer-carrying bacteria could express alternative S-layer protein genes for the adaptation to stressful environmental conditions (Sára and Sleytr, 2000; Jakava-Viljanen et al., 2002; Schär-Zammaretti et al., 2005; Grosu-Tudor et al., 2016). As shown by Grosu-Tudor et al. (2016), the S-layer composition of *L. acidophilus* IBB 801 was impacted by different culturing conditions like growth temperature, osmotic pressure, and pH. Probably, the homogenization induced a specific response passing through the activation of alternative *slp* genes that mediate the adaptation to the stress applied. In our work, HPH treatment increased bands between 45 and 50 kDa that could correspond to the Slp proteins, such as SlpX. In fact, Palomino et al. (2016) observed an increase in SplX upon osmotic stress. Since these speculations are based on MW profiles, more specific analyses on S-proteins, such as western-blotting or proteomics, would be needed. Other than the putative S-layer proteins, HPH treatment determined an overall increase of bands with high and low MWs. Tabanelli et al. (2015) showed an increase of low MW peptides in the HPH treated *L. acidophilus* DRU using a proteomic approach by means of MALDI-TOF. The generation of these molecules was attributed both to the effect of HPH on the cell-surface proteins and the cellular response to HPH treatment. In addition, an increase in the higher MW proteins could have been the result of both *de novo* protein synthesis and mechanical rearrangement of the outermost cell surface following the treatment applied. The discontinuous S-layer of the treated cells and the cell wall sublethal damages observed may have exposed proteins with hydrophobic regions that enhanced cell adhesion and hydrophobicity.

## Conclusion

High pressure homogenization applied at 50 MPa on *L. acidophilus* 08 improved the CSH (higher H%), autoaggregation and *in vitro* adhesion to mucin, compared to the untreated bacteria. The technology applied at a sublethal level also induced specific modulations on the general stress response and cell adhesion genes. In particular, stress response genes *groEL* and *ef-TU* were upregulated while *clpP* and the S-layer *slpA* gene were downregulated. Downregulation of *slpA* could have been compensated by a higher abundance of other Slp proteins. However, this hypothesis needs to be further studied in depth. A proteomic approach could be implemented to understand exactly which proteins are modulated during HPH treatment and to define which one could have an impact on the improved functionality. In fact, as reported by Wu et al. (2016), a comprehensive proteome profile of the target microbial species, when subjected to different stress, can provide a more reliable information in describing the molecular rescue strategy adopted by the strain to a specific stress. Eventually, to completely understand the entire HPH mechanism, the structure of peptidoglycan and LTAs following the HPH treatments needs to be elucidated. The results obtained in the present work may support the evidence that sublethal HPH treatments can positively affect some adhesion phenotypic traits of *L. acidophilus* 08 inducing a cell response between 60 and 120 min upon the treatment. Thus, the development of innovative strategies to increase strain probiotic performances by using the HPH technology should take into consideration the optimization of the operational protocols.

## Data Availability Statement

The original contributions presented in the study are included in the article/supplementary material, further inquiries can be directed to the corresponding author/s.

## Author Contributions

RL, GB, and FP designed the research, interpreted the data, and wrote the manuscript. GB and MD’A performed the research and analyzed the data. DG and LS contributed to the data interpretation. RL and FP supervised the experimental work and provided the funds. All authors contributed to revising the manuscript and approved the submitted version.

## Conflict of Interest

The authors declare that the research was conducted in the absence of any commercial or financial relationships that could be construed as a potential conflict of interest.
